# IDENTIFYING DIGITAL BIOMARKERS OF DEMENTIA-RELATED AGITATION: PRELIMINARY RESULTS FROM THE MODERATE STUDY

**DOI:** 10.1093/geroni/igz038.3333

**Published:** 2019-11-08

**Authors:** Wan-Tai M Au-Yeung, Lyndsey M Miller, Zachary Beattie, Christina L Reynolds, Jeffrey Kaye

**Affiliations:** Oregon Health & Science University, Portland, Oregon, United States

## Abstract

Agitation is a challenging dementia-related behavioral symptom and disconcertingly leads to an undesirable increase in pharmaceutical intervention. Agitation is primarily assessed subjectively by time-impoverished caregivers or clinicians, who may not be able to provide comprehensive or sensitive enough reporting to detect early signs of agitation or identify its precipitants. The purpose of the MODERATE (Monitoring Dementia Related Agitation Using Technology Evaluation) study is to characterize dementia-related agitation and its potential catalysts using wearables and sensors installed in participants’ private living quarters within memory care (skilled nursing) facilities. Preliminary data from seven episodes of agitation–reported by nursing staff and treated by pharmaceutical intervention–were captured and characterized with an integrated network consisting of research-grade actigraphy, passive infrared presence sensors, door contact sensors, ambient environmental sensor boards, and bed sensor mats. Behaviors (e.g. pacing, space transitions) during 8-hour nursing shifts that included episodes of agitation were compared with 8-hour nursing shifts without any episodes of agitation. The environmental conditions (temperature, lighting, humidity, and decibel level) during agitated periods and non-agitated periods were also compared. There was a statistically significant difference between agitated periods and non-agitated periods for the number of space transitions in the living room (p<0.01) and for the measured amount of ambient light and humidity (p <0.05). Passive monitoring of behavioral activity is a promising method of unobtrusive, early, and objective detection of dementia-related agitation, expanding the window of opportunity for identifying factors associated with behavioral change and delivering appropriate evidence-based interventions.

